# Notes and News

**Published:** 1904-10-15

**Authors:** 


### Notes and News.

Mr. John Langton has resigned the post of surgeon to St. Bartholomew's Hospital.

We understand that Mr. W. H. Cross, who has been clerk to St. Bartholomew's Hospital for 38 years, has sent in his resignation, and it is proposed to give him a pension of £900 per annum.

In a recent article on "The Hospital Penny Fund," which is being refloated in the office of *Public Opinion*, *Truth* practically challenged Sir Edward Walker and Sir Anthony Thornhill in respect to their connection with the scheme. Sir Anthony Thornhill has responded to the challenge. And, although he does not attempt to defend the countenance which he lends to a venture taking over 31 per cent. commission from the public for being so kind as to give their money for them to the hospitals, he explains that he "joined the committee at the request of a friend simply to look after his interests and to see on his behalf that the money which was ear-marked for distribution to the hospitals was properly administered." We can only conclude that Sir Anthony Thornhill does not know the cost of this "distribution," or else that he is quite unaware of what "properly administered" means.

Those who are of an observing turn of mind cannot but be struck with the shocking neglect of the teeth amongst the poor. Not infrequently almost toothless gums are seen, especially amongst women before the age of 30. We heard recently that amongst the mothers of the poor the loss of a tooth is a common occurrence with each addition to the family. Toothache is seldom regarded as a warning, and dentistry is looked upon as practically synonymous with tooth extraction. In Germany the knowledge of the importance of proper attention to the teeth has taken a practical form, and the municipal dentist is now an institution in German cities. In connection with some of the schools a regular inspection by a dentist is made, and any attention necessary is given. Russia is also moving in the same direction, and it is to be hoped that England will not be long behind. It is quite certain that when money is scarce the visit to the dentist is not likely to be entertained.

The last report from Mauritius announces 29 cases of plague with 27 fatalities.

The boatswain of the ship *Bishopsgate*, who was landed at Newcastle-on-Tyne, suffering from the plague, is now reported convalescent. The origin of the disease was traced to rats, and the boatswain evidently became infected when cleaning and disinfecting the ship at Hamburg.

Mrs. Bishop, the well-known author of travels, who died recently at Edinburgh, was also an active worker in the cause of the sick. She built no fewer than five hospitals in the East. She studied minor surgery and first aid in order to be of use on her travels. She is best known as an authoress under her maiden name of Bird.

The following entrance scholarships at the Charing Cross Hospital Medical School have been awarded:—The Epsom Scholarship (100 guineas) to Mr. L. H. Taylor, the Livingstone Scholarship (100 guineas) to Mr. C. J. Fox, the Huxley Scholarship (55 guineas) to Mr. H. F. L. Hugo, Universities Scholarships (each 72 guineas) to Mr. C. Beards and F. W. Wade. Entrance Scholarships have also been awarded to Mr. L. M. Webber (60 guineas), Mr. E. S. Calthrop (40 guineas) to Mr. R. G. Dainty (30 guineas), and an Universities Exhibition of 36 guineas to Mr. J. J. S. Rowe.

At the opening meeting of the London School of Tropical Medicine for the present Session, at which Sir John Craggs presided, Sir Charles Bruce delivered the inaugural address, and testified to the value of the school to the Empire. Sir Charles Bruce's great experience where malarial and tropical diseases prevail enabled him to speak with much weight on the subject. Sir P. Manson mentioned that the work was greatly crippled for want of funds, and said that £500 a year was needed for herbalological research. Sir Charles Bruce, in his address, laid stress upon the fact that the British Pharmacopœia was quite inadequate to meet the requirements of tropical disease.

Last year a committee of inquiry was appointed into the administration of the Salisbury Infirmary. One of the suggestions of this committee was that the present system of book-keeping should be brought into line with the uniform system, so that each item of expenditure might be classed under its proper head instead of many articles, as at present, being muddled up under dispensary, which really belong to provisions. In the annual report recently issued it is stated that Mr. Wilfrid Fletcher, who signs the accounts as a chartered accountant, is of opinion that it would be unwise to adopt the uniform system. Seeing that this system has been adopted with the approval of the leading firms of accountants by most of the great hospitals and many of the colonial Governments, the committee of management of the Salisbury Infirmary might usefully examine their auditor as to the reasons why he sets himself against the leading authorities in a matter of this kind. Mr. Wilfrid Fletcher's opinion is discounted by the fact that the committee of inquiry reported that the accounts kept under his supervision at Salisbury Infirmary did not properly analyse and classify the expenditure of that institution.

